# Global Analyses of Multi-Locus Sequence Typing Data Reveal Geographic Differentiation, Hybridization, and Recombination in the *Cryptococcus gattii* Species Complex

**DOI:** 10.3390/jof9020276

**Published:** 2023-02-20

**Authors:** Megan Hitchcock, Jianping Xu

**Affiliations:** Department of Biology, McMaster University, Hamilton, ON L8S 4K1, Canada

**Keywords:** *Cryptococcus*, multi-locus sequence typing, population genetics, phylogenetic analyses, geographic differentiation, hybridization, recombination

## Abstract

*Cryptococcus gattii* species complex (CGSC) is a basidiomycete haploid yeast and globally distributed mammalian pathogen. CGSC is comprised of six distinct lineages (VGI, VGII, VGIII, VGIV, VGV, and VGVI); however, the geographical distribution and population structure of these lineages is incompletely described. In this study, we analyze published multi-locus sequence data at seven loci for 566 previously recorded sequence types (STs) encompassing four distinct lineages (VGI, VGII, VGIII, and VGIV) within the CGSC. We investigate indicators of both clonal dispersal and recombination. Population genetic analyses of the 375 STs representing 1202 isolates with geographic information and 188 STs representing 788 isolates with ecological source data suggested historically differentiated geographic populations with infrequent long-distance gene flow. Phylogenetic analyses of sequences at the individual locus and of the concatenated sequences at all seven loci among all 566 STs revealed distinct clusters largely congruent with four major distinct lineages. However, 23 of the 566 STs (4%) each contained alleles at the seven loci belonging to two or more lineages, consistent with their hybrid origins among lineages. Within each of the four major lineages, phylogenetic incompatibility analyses revealed evidence for recombination. However, linkage disequilibrium analyses rejected the hypothesis of random recombination across all samples. Together, our results suggest evidence for historical geographical differentiation, sexual recombination, hybridization, and both long-distance and localized clonal expansion in the global CGSC population.

## 1. Introduction

Cryptococcosis is an infection caused by the inhalation of the human pathogenic *Cryptococcus* (HPC) from the environment. The opportunistic haploid yeast initially establishes within the lungs, and without treatment or in the case of long, asymptomatic latent periods, it can enter the bloodstream and into the central nervous system, leading to meningoencephalitis and potentially death [1]. Due to the enormous global burden, especially among patients with HIV infection [2], HPC was recently classified as a priority fungal pathogen by the World Health Organization (WHO). HPC is commonly found in nature, including in bird droppings, trees, and soils, all serving as environmental reservoirs of infectious propagules for humans [3,4,5,6]. HPC consists of two sister species complexes: the *Cryptococcus gattii* species complex (CGSC) and the *Cryptococcus neoformans* species complex (CNSC) [7].

For over 100 years, CGSC was referred to as *C. neoformans* var. *gattii* and treated as a variety of *C. neoformans* [8]. Because of this, *C. gattii* has often been overlooked and is most likely underrepresented in the available historical literature. It was Dr. June Kwon-Chung who emphasized the difference between isolates of *C. neoformans* and *C. gattii* and presented that *C. gattii* was predominantly found in tropical and subtropical climates, while *C. neoformans* was shown to be distributed globally [9,10]. Consistent with her observations, most of the early data regarding *C. gattii* came from regions of Australia and Papua New Guinea [11,12,13,14,15]. Early findings also suggested that *C. gattii* favored immunocompetent hosts, contrasting *C. neoformans*, which predominantly infects immunocompromised hosts [10]. 

*C. gattii* began attracting broader attention after an unusual outbreak started in the early 2000s on Vancouver Island, a large island off the coast of British Columbia, Canada. The outbreak has since spread to mainland BC and the Pacific Northwest in the U.S. [16,17]. Indeed, the frequency of *C. gattii* infection and colonization has increased dramatically in humans, animals, and the environment across these temperate regions [17,18,19,20]. Previously linked to eucalyptus trees [20,21,22], *C. gattii* has been isolated from soil, air, water, and >10 tree species native to Canada’s west coast [19]. In addition, the infection rate on Vancouver Island was found to be ~9 times higher than in the endemic region of Australia [23]. The proliferation of *C. gattii* in the temperate region as well as an increase in human populations have generated several hypotheses about its emergence and mechanism of dispersal. Surveys of air samples have found particles indictive of *C. gattii* spores [23], likely explaining its increasingly broad dispersals and high virulence, as spores are known to be more infectious than yeast cells [24]. In addition, basidiospores are produced from sexual reproduction, suggesting high levels of fertility among Vancouver Island isolates [25,26]. However, sampling also revealed isolates were nearly exclusively of mating type α [26], suggesting that opposite-sex mating for *C. gattii* in nature is likely uncommon [25]. In contrast, haploid fruiting and same-sex mating have been observed in *C. gattii*, both of which could have contributed to basidiospore production [27,28,29]. Indeed, evidence for recombination in nature has been reported for molecular type VGII within *C. gattii* [30]. This ability for mating and potential for meiotic recombination could also contribute to its adaptability, range expansion, and virulence. Together, these features of *C. gattii* call for greater efforts to understanding the epidemiology, pathogenesis, and evolution.

Although both CNSC and CGSC have a well-defined sexual phase [25], historically, it has been viewed as a predominantly clonal pathogen, with little evidence for sexual recombination in nature [31,32]. However, after fertile populations were reported on Vancouver Island, multiple studies were published regarding the mode of reproduction, where evidence for both clonal reproduction [33,34,35] and sexually recombination were present among defined populations in nature [36,37,38]. At present, the extent of recombination in the global populations of *C. gattii* remains unknown.

Since the 1990s, a variety of molecular methods has been used to identify the genotypes of strains of the human pathogenic *Cryptococcus*, including PCR fingerprinting [8], amplified fragment length polymorphism (AFLP) [39], multi-locus sequence typing [40], and whole-genome sequencing [41]. These studies have revealed the diversity and heterogeneity among *C. gattii* isolates and established that *C. gattii* is a species complex containing multiple distinct lineages. These lineages are frequently referred to as molecular types and increasingly more as species [42]. Formally, the *C. gattii* species complex is divided into six distinct lineages (VGI, VGII, VGIII, VGIV, VGV, and VGVI) [3,43,44]. In this report, we focus on the four lineages with isolates represented in the *International Fungal Multi Locus Sequence Typing* (IFMLST) database. Evidence for hybridization has been reported between some of the lineages within CGSC [30] as well as between CGSC and CNSC [45]. The extent of hybridization among the lineages and recombination within individual lineages in the global population of CGSC remains unknown.

To standardize genotyping and enhance data sharing and communication, in 2009, the International Society for Human and Animal Mycology (ISHAM) implemented the multi-locus sequence typing (MLST) scheme for global genotyping of CNSC and CGSC isolates. The proposed scheme recommends the use of partial DNA sequences from seven unlinked nuclear loci (*CAP59*, *GPD1*, *LAC1*, *PLB1*, *SOD1*, *URA5*, and *IGS1*) [46]. For each locus, all unique DNA sequences are assigned an allele type (AT), which in combination creates an allelic profile that is assigned a sequence type (ST). A publicly accessible database (The International Fungal Multi Locus Sequence Typing, IFMLST) stores the allele and sequence data for CNSC and CGSC [47]. This allows consistent and efficient comparisons across labs. Over the last 14 years, thousands of HPC isolates have been genotyped using the IFMLST scheme and with the data published and stored in the centralized website. The availability of this standardized genotyping method and genotype dataset enables global population genetic analyses of HPC. Indeed, our recent study of the *Cryptococcus neoformans* species complex revealed evidence for recombination and statistically significant geographic structuring of the two species within CNSC: *C. neoformans* and *C. deneoformans* [48].

In this study, we examined the global population structure of both the total CGSC samples and the individual lineages within CGSC by analyzing the published MLST data of this species complex. We extracted information from a total of 1202 non-redundant isolates from 31 published studies. We evaluated the overall geographical pattern of genetic variation as well as investigated potential evidence for recombination within individual lineages and hybridization among the lineages.

## 2. Materials and Methods

### 2.1. Strains, Genotypes and Metadata

We carried out a literature search through *PubMed* for the term “Cryptococcus MLST”. All papers were examined for relevant MLST data associated with isolates of CGSC, including *C. bacillisporus*, *C. gattii*, *C. deuterogattii*, and *C. tetragattii*, and all reported data regarding CGSC were retrieved and consolidated. All MLST data for strains of CGSC published by November 2022 were collected from the publicly available *International Fungal Multi Locus Sequence Typing* database. For each unique sequence type, the database stores its allelic profile and DNA sequences across seven loci. A total of 1202 isolates from 31 studies were included in our meta-analyses of population geographic structures. Protocols for DNA extraction, amplification, sequencing, and allelic profiling as well as detailed descriptions of these isolates and their genotype data can be found in the original reports [34,35,37,38,49,50,51,52,53,54,55,56,57,58,59,60,61,62,63,64,65,66,67,68,69,70,71,72,73,74,75].

### 2.2. Phylogenetic Distribution of Strains and Genotypes

To determine the relationships among STs within CGSC, DNA sequences at the seven MLST loci were concatenated for each sequence type. The concatenated sequences were then imported into MEGA X and aligned through Muscle [76,77] to reconstruct a phylogenetic tree using a neighbor-joining method based on the K2P distance model (Kimura 2-parameter model) [77]. Using the iTOL software [78], previously described lineage classifications from the IFMLST database were added to their associated ST to determine the distributions of the observed lineages in the overall phylogeny. Additionally, geographical and ecological source data for each ST found in the published literature were added to the specific ST to demonstrate their distributions on the phylogenetic tree.

### 2.3. Phylogenetic Distribution of Alleles

To investigate the partitioning of ATs among lineages, single-locus phylogenetic trees were reconstructed for all seven MLST loci using the DNA sequences for each AT in the IFMLST database. Three of the ATs associated with STs were excluded from the analysis because they did not have corresponding DNA sequences on the MLST database (AT91 at the *CAP59* locus) and (AT43 and AT44 at the *PLB1* locus). MEGA X software was used to reconstruct phylogenetic trees for the seven loci with neighbor-joining method based on muscle alignment and the K2P distance model (Kimura 2-parameter model) [76,77]. The trees were amended with metadata associated with each ST using the iTOL software [76]. In addition, the lineage data associated with the ST containing each AT was uploaded to the circular single locus trees to identify ATs shared across lineages.

### 2.4. Population Genetic Analyses

Population genetic analyses were performed to determine if the total CGSC and individual lineages within CGSC were genetically subdivided at the continental and country levels using GenAlEx V.6.5. [79]. Strains were subdivided based on the collected metadata and evaluated within five taxonomy-based samples: the complete CGSC sample, the VGI sample, the VGII sample, the VGIII sample, and the VGIV sample. For each taxonomic sample, both the clone-corrected (CC) and non-clone-corrected (NCC) datasets were analyzed. The CC dataset included only one isolate representing each ST, and the NCC dataset included all isolates retrieved from the literature.

An analysis of molecular variance (AMOVA) was conducted for each of the ten datasets (five taxonomic CC datasets and five taxonomic NCC datasets) at the country and continental levels. Additionally, Wright’s F_ST_ values between pairs of geographical populations were determined. To prevent potential biases due to small sample sizes, only subpopulations with >five isolates were included in these analyses. Statistical significance was determined by comparing the observed results with 1000 permutated datasets established with a null hypothesis of no genetic differentiations among geographic populations within each analyzed dataset.

Phylogenetic compatibility and linkage disequilibrium analyses were then conducted using the clone-corrected allelic profiles at the seven loci across 566 STs to test for evidence of recombination within and among individual lineages. Tests were performed across 17 population samples: the total CGSC sample, the four CGSC lineages (VGI, VGII, VGIII, and VGIV) at the global level, the CGSC sample divided by each continent (Africa, Asia, Europe, Oceania, South America, and North America), as well as six population samples of individual lineage by continent combinations for samples with more than 20 recorded STs (Asia VGI, Europe VGI, Oceania VGI, North America VGI, North America VGII, and South America VGII). Information regarding the underlying concepts of these analyses and how they were conducted can be found in the MultiLocus V1.3 manual [80].

## 3. Results

As of November 2022, there were 566 sequence types (STs) with affiliated DNA sequence data for all seven loci stored on the Cryptococcus MLST database for CGSC. Geographical data were available for 375 STs (66%) representing 1202 isolates collected from 31 published reports along with associated metadata. However, the remaining 191 STs (33%) were not associated with any geographic information. Our population genetic analyses focused on the 375 STs with geographic information. However, for recombination and hybridization analyses, all STs were included. Below, we outline the collected data regarding the 1202 isolates and illustrate the results of our analyses.

### 3.1. Geographical and Ecological Distributions

The 375 STs with geographic information representing 1202 isolates were subdivided into four previously proposed lineages (VGI, VGII, VGIII, and VGIV) as indicated by original authors. Among these four lineages, VGII was the most prevalent (721 isolates; 60%), followed by VGI, VGIII, and VGIV, containing 225 (19%), 221 (18%), and 34 (3%) isolates, respectively. The CGSC isolates as well as lineages VGI and VGII were distributed across all six continents. However, among the isolates in the MLST database, VGIV was not represented in North America, and VGIII was not recorded in Africa or Asia. In total, the CGSC isolates were distributed across 45 countries. Among the four lineages, VGI was recorded from the most countries (31), followed by VGII (25), VGIII (12), and VGIV (9). Out of the 1202 isolates with geographical data, only 788 isolates (65.5%) included ecological source data, while the remaining 414 isolates (34.5%) were not associated with any source information. Here, we broadly categorize the isolates into three ecological sources: clinical, environmental, and veterinary. Among the 788 isolates with ecological source information, 416 were collected from clinical sources (53%), followed by environmental (259 isolates; 33%) and veterinary (113 isolates; 14%) sources (Supplementary Table S1). The total CGSC, VGII, and VGIII were reported from all three sources, but VGI was not recorded from veterinary sources, and VGIV was not recorded from environmental sources. Further details pertaining to ecological source data regarding the four lineages is provided in Supplementary Table S2 and summarized in Table 1.

The geographical distribution of the non-redundant 1202 CGSC isolates as well as the isolates subdivided into the four lineages are shown in Table 2. These 1202 isolates were recorded across six continents and 45 countries. At the species complex level, most CGSC isolates in the database were from South America (517; 43%), followed by Asia (196; 16%), North America (179; 14.9%), Oceania (159; 13%), Africa (86; 7%), and Europe (65; 5%). Among the lineages, VGII was the most prevalent across South America, Asia, and Africa. However, VGIII was the most prevalent in North America and VGI the most prevalent in Europe. At the country/region level, most isolates were recorded from Brazil (349; 29%), followed by Australia (153; 12.7%), Colombia (145; 12%), the USA (141; 11.7%), and Taiwan (111; 9%). The remaining 38 countries each contained <100 isolates and together were represented by 303 of the isolates in the published literature.

Table 3 summarizes the geographic and ecological distributions of the 375 STs in the published MLST literature for CGSC with associated metadata. Geographically, among the 375 STs, none were reported across all six continents or a combination of five continents. Only three STs (representing 61 isolates) were each reported from four of the six continents, seven STs (representing 254 isolates) were each reported from three of the six continents, and twenty-four STs (representing 226 isolates) were each reported from two of the six continents. The remaining 341 STs (representing 658 isolates) were reported in only one of the continents. Among the continents, the smallest number of STs were reported from Africa (24 STs representing 32 isolates), and the largest was from South America (156 STs representing 331 isolates) (Table 3). Ecologically, among the 375 STs with geographic information, 188 had ecological niche data. Of these 188 STs, 5 (representing 237 isolates) were each reported from all three ecological niches, 17 (representing 205 isolates) were each reported from two of the three ecological niches, and 166 (representing 346 isolates) were each reported from only one ecological niche. The majority, 123 STs (representing 255 isolates), were reported from only clinical sources (Table 3). The detailed geographic associations of all 375 STs at the continental and country/region levels are presented in Supplementary Tables S3 and S4, respectively.

### 3.2. Geographic AMOVA

The overall objective of our AMOVA analyses was to determine the amount of genetic variation contributed by geographic separations at the continental and country/region levels. Below, we briefly summarize the results. At the continental level analyses, in the non-clone-corrected sample, genetic variations within continents contributed 83%, 79%, 84%, 88%, and 92% of the total observed genetic variations in the total CGSC population, VGI population, VGII population, VGIII population, and VGIV population, respectively. The remaining 17%, 21%, 16%, 12%, and 8% of the genetic variations were due to among continents. The within-continent and among-continent contributions for four of the populations (total CGSC, VGI, VGII, and VGIII) were statistically significant at the *p* < 0.001 level, and the VGIV population was statistically significant at the *p* < 0.05 level (Table 4). In the five clone-corrected samples, genetic variations within continents contributed 95%, 85%, 96%, 97%, and 97% of the total observed genetic variations in the entire CGSC population, the VGI population, VGII population, VGIII population, and VGIV population, respectively, all greater percentages than those without clone corrections. The remaining 5%, 15%, 4%, 3%, and 3% of the total genetic variations were attributed to among continents. The among-continent contributions for three of the five populations (total CGSC, VGI, and VGII) were statistically significant at *p* < 0.001, while for the VGIII and VGIV populations, they were not statistically significant, with *p* = 0.07 and *p* = 0.2, respectively (Table 4). The pairwise comparisons between continents for the five taxonomic samples are shown in Table 5. In addition, variable numbers of pairwise country/region level comparisons also showed statistically significant genetic differentiations. The detailed pairwise country/region level comparisons for the total CGSC, VGI, VGII, VGIII, and VGIV samples are presented in Supplementary Tables S5, S6, S7, S8, and S9, respectively.

### 3.3. Ecological Niche AMOVA

Further AMOVA analyses were conducted to determine the amount of genetic variation contributed by ecological niche separation within five countries/region each with a sample size greater than 100 (Australia, Brazil, Colombia, the USA, and Taiwan). Both the non-clone-corrected and clone-corrected populations were analyzed and divided into clinical, environmental, and veterinary sample populations. Only ecological populations with >9 isolates were considered. Because of the small sample size, the clone-corrected Australia and clone-corrected Taiwan populations were not analyzed. The remaining three clone-corrected sample populations (Brazil, Colombia, and the USA) failed to reject the null hypothesis that ecological niche separations contributed significantly to the total observed genetic variation within each country. However, all five non-clone-corrected population samples rejected the null hypothesis of no genetic differentiation among ecological samples at the *p* < 0.001 level, representing 22%, 11%, 10%, 13%, and 10% of genetic variation coming from among ecological niches in Australia, Brazil, Colombia, the USA, and Taiwan, respectively. These results are summarized in Table 6, and the pairwise comparisons are demonstrated in Supplementary Table S10.

### 3.4. DNA Sequence Variation

The allelic profiles of all 566 STs, including the allele type (AT) number at each of the seven MLST loci (*CAP59*, *GPD1*, *LAC1*, *IGS1*, *PLB1*, *SOD1*, and *URA5*) for each ST were retrieved from the IFMLST database. A summary of the total reported ATs for each of the seven loci as well as the proportion of shared ATs across the four VG lineages is shown in Table 7. The AT numbers among the seven loci ranged from 46 at the *PLB1* locus to 136 at the *SOD1* locus. In addition, the proportion of alleles shared among the four VG lineages ranged from 1 to 6, with the *GPD1* locus containing the most shared alleles (6) and loci *CAP59*, *PLB1*, and *URA5* each having only one shared allele. The differences in length (of base pair) among ATs at each locus ranged from 2 bp for *CAP59* to 82 bp for *IGS1.* The range of occurrence of each AT within each of the four lineages is shown in Supplementary Table S11.

### 3.5. Phylogenetic Analysis

**Figure 1:**
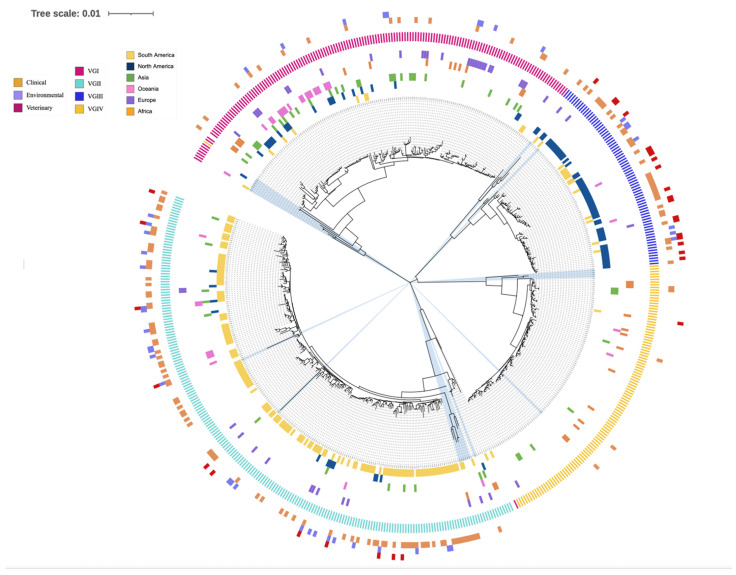
Phylogenetic tree illustrating the relationship among 566 sequence types (ST) of the *C. gattii* species complex. The tree was constructed using the neighbor-joining method based on K2P distance in MEGA11. The branch lengths are proportional to sequence divergence among sequence types. Three large circles and ten small circles are used to display the association of metadata with each ST. The outermost large circle, containing three small circles, demonstrates the ecological niche each ST was isolated from, with each color representing a specific niche (clinical, environmental, and veterinary). The middle large circle contains only one circle, displaying four colors, each representing one of the four CGSC lineages (VGI, VGII, VGIII, and VGIV). The inner most circle, containing six smaller circles, displays the continental distribution of each ST, with each color representing one continent (Africa, Asia, Europe, Oceania, North America, and South America). Legends on the left indicate the correspondence between each color and the associate metadata for each ST. STs shaded in blue contain alleles shared between more than one lineage.

Phylogenetic investigations of the concatenated sequences at the seven MLST loci showed four clearly separated major ST groups, largely corresponding to four VG lineages. However, two STs (ST402 and ST351) were grouped inconsistently to the lineages that they were originally assigned in the published reports. In addition, two STs with previously undefined lineages (ST2 and ST71) were clearly grouped into VGI and VGIV, respectively (Figure 1).

**Figure 2:**
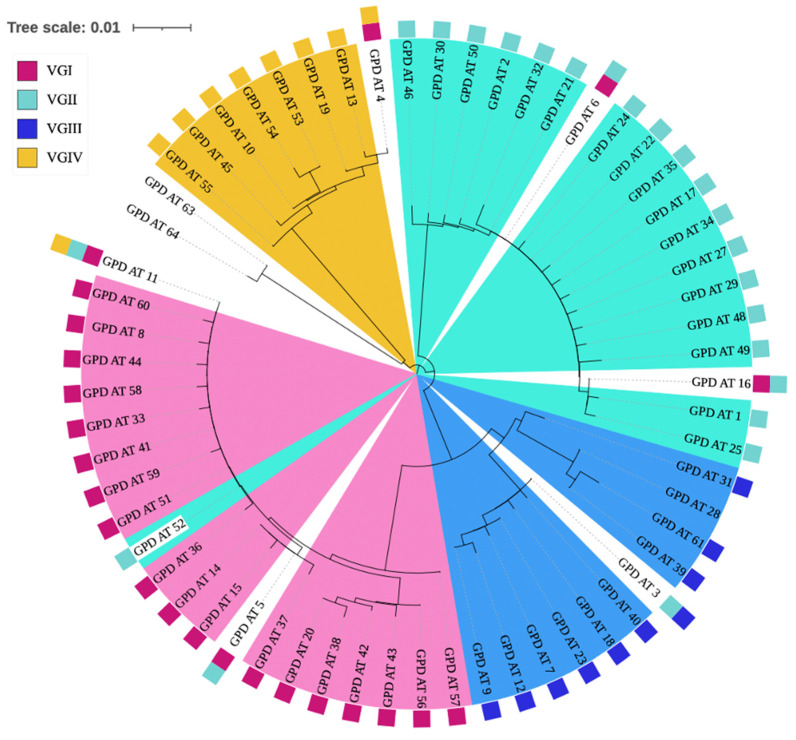
Phylogenetic tree illustrating the relationship among 61 allele types (AT) found at the *GPD1* locus of the *C. gattii* species complex. The tree was constructed using the neighbor-joining method based on K2P distance in MEGA11. The branch lengths are proportional to sequence divergence among allele types. Four colors are used to display in which lineage each AT is recorded. Legend on the left indicates which color corresponds to which lineage (VGI, VGII, VGIII, and VGIV). Six of the sixty-one ATs were associated with STs belonging to at least two different lineages.

Phylogenetic analyses of the single-locus trees were overall consistent with the phylogenetic pattern based on concatenated gene sequences. However, there was several interesting observations. First, 1–2 allele types at each of the seven loci showed great divergence from their expected clusters. These included AT96 at the *CAP59* locus, AT63 and AT64 at the *GPD1* locus, AT64 at the *LAC1* locus, AT46 and AT47 at the *PLB1* locus, AT113 at the *IGS1* locus, AT133 and AT134 at the *SOD1* locus, and AT59 at the *URA5* locus. Single-locus phylogenetic trees including all ATs can be seen in Figure 2 (for locus *GPD1*) and Supplementary Figures S1–S6 (for other six loci). ATs across five of the seven single-locus trees (*CAP59*, *LAC1*, *PLB1 IGS1*, and *SOD1)* showed no inconsistencies between expected and observed VG lineage clustering. However, the *GPD1* and *URA5* single-locus trees contain one and two instances of inconsistent VG lineage grouping. At the *GPD1* locus, AT52 (as part of ST400 and ST409) was expected to be grouped within the VGII cluster based on their original assignment in the database but instead was placed within the VGI cluster. At the *URA5* locus, AT35 (as part of ST336) and AT34 (as part of ST419) were both grouped within the VGII cluster but were expected to be within the VGIII and VGIV clusters, respectively.

### 3.6. Inconsistent Allele Type Clustering

Interestingly, each of the seven single-locus trees included at least one occurrence of shared alleles among VG lineages representing potential signatures of recombination or loss of heterozygosity after hybridization. In total, of the 566 STs, 23 (4%) contained an unexpected allele shared with a VG lineage not associated with their originally proposed lineage. Interestingly, ST402 and ST351, which were inconsistently grouped in Figure 2, include six and four shared alleles, respectively. Out of the consistently grouped STs, two (ST431 and ST524) contained two shared alleles each, involving a total of three VG lineages. The remaining 19 STs each contained at least one allele shared between two VG lineages. The complete allelic profiles of these 23 STs are displayed in Table 8.

### 3.7. Recombination and Linkage Disequilibrium

We examined potential indicators of recombination by investigating two standard signatures: phylogenetic incompatibility and linkage equilibrium. Here, we analyzed a total of 17 population samples including the total CGSC population and the four major VG lineages (VGI, VGII, VGIII, and VGIV) at the global level, the total CGSC sample separated by continent (Africa, Asia, Europe, Oceania, North America, and South America), as well as individual lineages separated by continent for populations >20 STs (Asia VGI, Europe VGI, Oceania VGI, North America VGI, North America VGII, and South America VGII). In the phylogenetic incompatibility test, we found that none of the 17 population samples showed 100% phylogenetic compatibility (Table 9). Five of the seventeen population samples (Global CGSC, Global VGI, Global VGII, South America CGSC, and South America VGII) explicitly showed no phylogenetic compatibility among the seven loci. This was consistent with evidence of recombination at the global level among 21 pairs of loci within each of these three samples. VGIII showed 19 of the 21 pairwise loci to be phylogenetically incompatible, while VGIV revealed 16 of 21 pairwise loci to be phylogenetically incompatible. Together, the phylogenetic compatibility test showed that all four VG lineages contained evidence for recombination at the global level. The continental-level analyses displayed much lower frequencies of phylogenetic incompatibility for all continents except South America, which remained consistent for strong evidence for a highly recombing population.

At the global level, linkage disequilibrium analyses revealed that in all five samples, the null hypothesis of random recombination was rejected (Table 9). However, within each of the five samples, variable numbers of pairs of loci showed no significant deviation from those expected under the random recombination null hypothesis. For example, in the global VGI sample, 10 of the 21 loci pairs had observed genotype frequencies not significantly different from random recombination, all involving the *CAP59* or *IGS1* loci. Similarly, in the global VGIV sample, 5 of the 21 loci pairs had observed genotype frequencies not significantly different from random recombination, all involving the *GPD1* locus. In comparison, no evidence for linkage equilibrium across all 21 loci pairs was observed within the total global CGSC sample, the global VGII sample, and the global VGIII sample. The complete allelic associations among loci for all 17 samples are shown in Supplementary Table S12.

## 4. Discussion

This study extracted MLST data of CGSC from 31 peer-reviewed publications and the IFMLST database. The IFMLST database currently consists of 566 established sequence types (STs) grouped into four VG lineages (VGI, VGII, VGIII, and VGIV) representing the recognized MLST genotypes of CGSC. The extracted MLST data were used to investigate the population structure of the total CGSC and each of the four lineages within CGSC. Below, we discuss the results of our analyses.

### 4.1. VG Lineage Distribution

As reported in the original literature [34,35,37,38,49,50,51,52,53,54,55,56,57,58,59,60,61,62,63,64,65,66,67,68,69,70,71,72,73,74,75], each of the four CGSC lineages (VGI, VGII, VGIII, and VGIV) in the IFMLST database are broadly distributed across multiple continents. However, the four lineages showed different patterns: the VGI lineage was recorded in 30 of the 45 countries with CGSC, followed by VGII (in 25 countries), VGIII lineage (in 11 countries), and VGIV lineage (in 9 countries). This summarized distribution is consistent with past literature illustrating a greater worldwide distribution of the VGI and VGII lineages compared to the VGIII and VGIV lineages [55]. Among these four lineages at the global level, VGII was the most abundantly represented in the MLST database and was higher than VGI lineage, which was often considered as the most abundant lineage worldwide [55]. However, within Europe as well as in 15 of 45 countries/regions, VGI lineage was the most prominent. Congruent with our global results in the published MLST literature, the VGII lineage was the most prevalent within Africa, Asia, Oceania, and South America as well as in 15 of 45 countries. The VGIII lineage was the most prominent within North America as well as in 6 of 45 countries. This was unexpected, as the VGII lineage is credited with causing the outbreak across Vancouver Island and the Pacific Northeast [23]. Indeed, excluding isolates associated with this outbreak, the VGIII lineage was the most abundant in North America. Such a pattern was used as evidence for an environmental shift of the VGII lineage beyond tropical and sub-tropical regions into temperate North America [81]. Our meta-analyses identified no isolate from the VGIII lineage within Africa or Asia, which is consistent with previous studies that found no isolate of the VGIII lineage in China [56,61]. The VGIV lineage was the most abundant in four countries, including a clonal expansion of ST173 representing 43 isolates in Ivory Coast [81,82,83].

### 4.2. Geographic and Ecological Structuring

Our global population genetic analyses included 1202 isolates representing 375 STs from 45 countries across six continents. Several STs were broadly distributed, consistent with long-distance clonal expansion of these STs across countries and continents. However, of the 375 STs, 341 STs (90%) were sampled from a single continent, and none were recorded from all six or a combination of five continents. This pattern is different from what was observed in the sister-species complex CNSC, where several STs (ST5 and ST93) were broadly and abundantly distributed [48]. While a lack of CGSC sampling might have contributed to the observed pattern, our population analyses revealed statistically significant differentiations among continental and national populations of CGSC, suggesting relatively limited gene flow across the globe. In the total CGSC sample, the observed genetic differentiations were mostly due to variations in the distribution of CGSC lineages as discussed in the last section as well as localized clonal expansion of certain STs. Indeed, clonal expansion of specific STs was found for all lineages, most of which were localized to individual or subset of countries and continents. For instance, the most abundantly collected ST in VGI was ST106, represented by 37 isolates; in VGII, it was ST7, represented by 167 isolates; in VGIII, it was ST9, represented by 27 isolates; and in VGIV, it was ST336, represented by 5 isolates, and all were predominantly found within one to a few countries. Localized clonal expansion can potentially bias the allele and ST frequencies, contributing to the observed genetic difference among geographic populations [84]. Because of this, we further analyzed clone-corrected samples where only one representative from each geographical region was included. Using clone-corrected samples, the total genetic variance among continents was reduced by 12%, 6%, 12%, 9%, and 5% across the total CGSC population, VGI lineage, VGII lineage, VGIII lineage, and VGIV lineage, respectively. In addition, using clone-corrected samples, the total genetic variance among countries was reduced by 8%, 17%, 23%, 10%, and 12% across the total CGSC population, VGI lineage, VGII lineage, VGIII lineage, and VGIV lineage, respectively. While the contributions by geographic separation were reduced in the clone-corrected samples, in both the total CGSC as well as the VGI and VGII lineages, statistically significant genetic differentiations were still observed and were consistent with the existence of endemic genetic variations within most regional populations. Overall, these results suggest greater evidence for geographical structuring and less evidence for global gene flow in CGSC than in *C. neoformans* species complex [48].

Ecological data were available for 788 isolates represented by 188 STs. Across the whole CGSC population, VGI lineage, VGII lineage, VGIII lineage, and VGIV lineage, most isolates (416, 52, 284, 70, and 9, respectively, for the five sample types) were sampled from a clinical source, followed by (259, 51, 172, 36, and 0, respectively) from environmental sources, and the remaining (113, 0, 74, 38, and 1, respectively) from veterinary sources. The overall prevalence of isolates recorded from clinical settings is likely due to a greater effort in obtaining and analyzing clinical specimens than from environmental and veterinary sources. Because patient infection by CGSC is from environmental sources, it is important that we increase our efforts to understand environmental populations of this pathogen and identify the potential factors that may contribute to the differences between clinical and environmental samples and how factors in the environment such as fungicide usage may impact clinical antifungal resistance in CGSC [85]. Indeed, our analyses of ecological samples within each of five countries/regions with >100 isolates of CGSC showed statistically significant genetic differentiation in the non-clone-corrected samples but limited differences in the clone-corrected samples. Such results are consistent with the overall similarities among ecological samples in their allelic and sequence types at the seven loci within each of the five countries/regions but that the different ecological niches had clonally expanded frequencies of different allelic and sequence types. Our results suggest that most environmental CGSC alleles and sequence types are capable of infecting humans and animals, but they likely differ in their virulence. Similarly, many human fungal pathogens capable of infecting animals may also serve as a reservoir for the evolution of virulent strains to humans. Understanding the relationships among the ecological populations could have significant implications in our understanding of the evolution of virulence and drug resistance in fungal pathogens [86]. Indeed, in CGSC, genotype sharing among strains from the three different sources were common [Table 3]. For example, CGSC was transferred to a new region within Australia by transporting infected koalas [75]. Similarly, the dispersal of CGSC related to the Vancouver Island outbreak was facilitated by tree cutting and transportation of trees and wood products [87].

### 4.3. Hybridization and Recombination

We performed phylogenetic analyses using the published 566 STs from the IFMLST database to examine the overall population structure of *C. gattii* species complex using distance-based models. Overall, the phylogenetic results were consistent with the four main lineages as revealed based on PCR fingerprinting, AFLP, and/or PCR-RFLP of the *URA5* gene fragment. There were two cases of inconsistent observed grouping: ST351 and ST402 observed in Figure 1. ST351 was previously designated within the VGI lineage [47] and contains ATs at the *CAP59*, *IGS1*, and *URA5* loci within VGI. However, the concatenated sequence of ST351 was grouped within the VGII branch and with ATs at the remaining four loci *GPD1*, *LAC1*, *PLB1*, and *SOD1* clustered within the VGII lineage. ST402 was previously designated within the VGIV lineage [47] but contains only the AT at the *URA5* locus within the VGIV cluster. Interestingly, ST402 contains AT at the *PLB1* locus being grouped within the VGII lineage, while alleles at the remaining five loci were clustered within the VGI lineage. The dominance of VGI alleles (5/7) within ST402 led to the clustering of ST402 within the VGI branch. In most cases, lineage designation of strains of human pathogenic *Cryptococcus* was derived based on PCR-restriction fragment length polymorphism at the *URA5* locus [88]. Aside from ST351 and ST402, our analyses identified 21 other STs with ATs from two or more lineages. Together, these results are consistent with hybridization involving all four lineages within CGSC. Based on sequence polymorphisms in the mitochondrial genomes, previous studies showed evidence of hybridization between VGI and VGII lineages [30] and between VGII and VGIII lineages [89]. In addition, both mitochondrial and nuclear genome recombination were suggested within the VGI and VGII lineages [30,33,37]. Though most isolates were classified correctly to individual lineages based on this method, our analyses here suggest that caution should be taken when using the PCR-RFLP results at the *URA5* locus for lineage assignment.

In addition to hybridizations among lineages, we identified evidence for recombination within each of the four lineages. The results are consistent with sexual reproduction playing a role in natural populations of CGSC. However, some of the observed phylogenetic incompatibility could be due to reverse mutations, convergent evolution, and/or incomplete lineage sorting. Additional investigations are needed to determine whether some of the nucleotide substitutions at the seven MLST loci have been positively selected and potentially contributed to the observed phylogenetic incompatibilities (and hence recombination). Similar to those observed for CNSC [48], evidence for asexual reproductions were prevalent in CGSC. Asexual reproduction enables rapid colonization of a suitable habitat [90] and spread of an infectious clone while sexual recombination increases the likelihood of generating a diversity of genotypes and potentially facilitating population adaptation to changing environments [91]. Clonality and sexual reproduction are not mutually exclusive, and frequent studies have documented cases of both in many fungal species [52,63,69,92,93]. In CGSC, one report illustrated a prevalence for clonal dispersal in Cucuta, Colombia [59], while another study reported an exceptionally diverse population with evidence for sexual recombination across multiple regions of Colombia [37]. Similarly, a study that looked at STs associated with the Vancouver Island outbreak (ST20 and ST7) concluded there was significant evidence for both clonal expansion and sexual reproduction within the VGII lineage [69].

Several theories have been brought forward to illustrate the potential origin(s) of the Vancouver Island outbreak thought to involve ST20 and ST7 [35,72], with most involving a mixture of clonal expansion and sexual recombination [23,26]. ST7 is a globally distributed sequence type [47]. For instance, 11 out of 12 CGSC isolates identified in Thailand were identical to ST7 associated with the Vancouver outbreak [35], and another report credited clonal expansion of ST7 as responsible for an ongoing VGII outbreak throughout Australia [34]. Our study recorded 167 isolates of ST7, making it the most prevalent ST of CGSC globally, represented across 10 countries/regions (Australia, Brazil, Canada, China, Colombia, South Korea, Taiwan, Thailand, and the USA), demonstrating a global presence of a clonally dispersed ST. In contrast, ST20 is endemic to the Amazon region [72] and was shown to display a large, scattered distribution within the region [39]. Our study recorded 40 cases of ST20, 31 of which were sampled from Brazil. A recent study showed evidence for high rates of recombination within the Brazilian VGII population and suggested that the Vancouver Island outbreak might have emerged from the Brazilian population, followed by clonal dispersal [38,94].

*C. gattii* species complex has a known sexual phase with two mating types, *MAT**a*** and *MAT****α***, defined at the mating type (*MAT*) locus [95,96]. In the case of opposite-sex mating, meiotic progeny is expected to have even proportions of the two mating types. However, that is not typically observed in nature [33]. Reports predominantly describe a *MAT****α***-dominant population within CGSC and the sister species CNSC. However, some populations, specifically in Australia, have been described as predominantly *MAT**a*** [97]. Since the *MAT* locus is not included within the MLST scheme, this study did not account for the distribution of *MAT**a*** versus *MAT****α*** strains in our analyses. Nonetheless, it seems uncommon to observe an equal distribution of both mating types in nature. Evidence suggesting sexual recombination within populations with a bias mating type can be explained by same sex mating [23,27,96]. This has been extensively discussed in the Vancouver Island outbreak population [27].

Sexual mating and reproduction among lineages have been observed in the lab. However, most hybrid progeny in laboratory crosses were diploid/aneuploid, with evidence of heterozygosity across most chromosomes [98]. In contrast, the observed hybrids were homozygous at all seven loci. The lack of heterozygosity could be achieved through a series of loss of heterozygosity (LOH) events during mitotic divisions after the initial hybridization [99]. Hybridization has also been recorded between isolates of HPC sister species CGSC and CNSC as well as between distinct lineages (*C. neoformans* and *C. deneoformans*) of CNSC [45,100]. Due to the distinct chromosomal structures of different lineages within HPC, meiosis in hybrids among lineages is often incomplete, and diploid or aneuploid sterile offspring are often produced [100]. During subsequent clonal budding, the hybrids could lose heterozygosity, resulting in recombinant haploid yeast cells that are genetically different from both parental strains. Together, these results suggest that although the CGSC population contains historically differentiated lineages, there is ongoing genetic exchange within and among these lineages, potentially generating distinct genotypes that are highly adaptive and virulent to humans.

In conclusion, our meta-analyses of the published MLST data allowed us to determine the genotype distribution and population structure of the *C. gattii* species complex. Our analyses illustrated geographic structuring of the CGSC population. While evidence for clonal dispersal was found, there is evidence for significant genetic differentiations in all four lineages at both the continental and country levels, with localized clonal expansion contributing to the observed geographic differences within most lineages. However, evidence for 19 incidences of ATs shared across VG lineages and significant evidence for recombination within all lineages demonstrates that genetic exchanges within and among lineages are ongoing. Continued population monitoring of CGSC, especially from environmental sources, is needed in order to effectively cope with the threats caused by this deadly pathogen. 

## Figures and Tables

**Table 1:** Summary lineage distribution of strains of the *C. gattii* species complex genotyped using the ISHAM multi-locus sequence typing scheme.

Sample Group	No. of Recorded ST	No. of ST with Geographical Data	No. of Isolates with Geographical Data	Found in No. of Continents	Found in No. of Countries	No. of ST with Ecological Data	No. of Isolates with Ecological Data	Found in No. of Ecological Sources
Total CGSC	566	375	1202	6	45	188	788	3
VGI	160	97	225	6	31	23	103	2
VGII	218	181	721	6	25	105	530	3
VGIII	75	72	221	4	12	56	144	3
VGIV	111	24	34	5	9	7	10	2
ST previously unassigned to a lineage	2	1	1	1	1	1	1	1

No., number; Ecological sources are defined as: clinical, environmental, and veterinary.

**Table 2:** Summary geographic distributions of isolates of the *C. gattii* species complex genotyped using the ISHAM multi-locus sequence typing scheme.

Region	*n*	%	VGI	VGII	VGIII	VGIV	Region	*n*	%	VGI	VGII	VGIII	VGIV
**Africa**	86	7.15%	20	45	0	21	**North America**	179	14.89%	25	15	139	0
Ivory Coast	45	3.74%	2	43	0	0	USA	141	11.73%	20	7	114	0
DRC	12	1.00%	10	2	0	0	Mexico	25	2.08%	1	0	24	0
South Africa	12	1.00%	4	0	0	8	Canada	10	0.83%	3	7	0	0
Zimbabwe	12	1.00%	0	0	0	12	Aruba	1	0.08%	0	1	0	0
Rwanda	3	0.25%	2	0	0	1	Cuba	1	0.08%	1	0	0	0
Kenya	2	0.17%	2	0	0	0	Guatemala	1	0.08%	0	0	1	0
**Asia**	196	16.31%	87	141	0	9	**Oceania**	159	13.23%	24	129	4	2
Taiwan	111	9.23%	44	66	0	1	Australia	153	12.73%	20	129	2	2
China	43	3.58%	28	14	0	0	New Zealand	3	0.25%	1	0	2	0
Thailand	17	1.41%	3	14	0	0	Papa New Guinea	3	0.25%	3	0	0	0
India	10	0.83%	6	0	0	4							
Malaysia	10	0.83%	3	3	0	4							
Singapore	3	0.25%	3	0	0	0							
Japan	1	0.08%	0	1	0	0							
South Korea	1	0.08%	0	1	0	0							
**Europe**	65	5.41%	44	17	3	1	**South America**	517	43.01%	25	416	75	1
Spain	14	1.16%	12	1	1	0	Brazil	349	29.03%	9	340	0	0
Greece	11	0.92%	8	3	0	0	Colombia	145	12.06%	13	63	68	1
France	9	0.75%	5	4	0	0	Argentina	10	0.83%	1	7	2	0
Italy	9	0.75%	9	0	0	0	Venezuela	5	0.42%	0	3	2	0
The Netherlands	8	0.67%	2	6	0	0	Paraguay	2	0.17%	0	0	2	0
Germany	5	0.42%	2	1	2	0	Peru	2	0.17%	2	0	0	0
Portugal	3	0.25%	3	0	0	0	Uruguay	2	0.17%	0	2	0	0
Belgium	2	0.17%	2	0	0	0	Chile	1	0.08%	0	0	1	0
Switzerland	2	0.17%	1	1	0	0	French Guiana	1	0.08%	0	1	0	0
Denmark	1	0.08%	0	1	0	0							
Sweden	1	0.08%	0	0	0	1							

***n***, total isolates; %, percent of isolates of the total recorded in CGSC.

**Table 3:** Summary distribution of 375 sequence types of the *C. gattii* species complex among continents and ecological niches.

Distribution Patterns	Specific Continent(s)/Ecological Niche(s)	Number of Sequence Types	Number of Isolates
Geographic			
In all six continents	Africa + Asia + Europe + N America + S America + Oceania	0	0
In five continents only		0	0
In four continents only			
	Asia + Europe + N America + S America	2	50
	Asia + Europe + N America + Oceania	1	11
	Other combinations of four continents	0	0
In three continents only			
	Africa + Asia + Europe	3	56
	Asia + Europe + S America	1	8
	Asia + N America + S America	2	186
	Asia + N America + Oceania	1	4
	Other combinations of three continents	0	0
In two continents only			
	Africa + Asia	1	2
	Africa + Europe	1	3
	Africa + S America	1	3
	Asia + Europe	2	5
	Asia + N America	3	41
	Asia + S America	3	42
	Asia + Oceania	1	4
	Europe + N America	2	4
	Europe + S America	2	8
	N America + S America	5	95
	N America + Oceania	2	6
	S America + Oceania	1	16
	Other combinations of two continents	0	0
In one continent only			
	Africa	24	32
	Asia	30	74
	Europe	31	45
	N America	75	131
	S America	156	331
	Oceania	25	45
Ecological niches			
In all three niches		5	237
In two niches only			
	Clinical + Veterinary	5	31
	Clinical + Environmental	10	165
	Veterinary + Environmental	2	9
In one niche only			
	Clinical	123	255
	Veterinary	19	22
	Environmental	24	69

**Table 4:** Analysis of molecular variance at the continental level.

	Non-Clone-Corrected					Clone-Corrected				
	df	MS	Est.Var	%	*p*-Value	df	MS	Est.Var	%	*p*-Value
Total CGSC										
Among continents	5	98.373	0.540	17%	0.001	5	14.283	0.175	5%	0.001
Within continents	1196	2.646	2.649	83%	0.001	417	3.100	3.100	95%	0.001
Total	1201	3.045	3.189			422	3.23	3.275		
VGI										
Among continents	5	20.456	0.532	21%	0.001	5	10.329	0.408	15%	0.001
Within continents	219	2.050	2.050	79%	0.001	115	2.264	2.264	85%	0.001
Total	224	2.46	2.582			120	2.6	2.672		
VGII										
Among continents	4	46.516	0.417	16%	0.001	4	4.798	0.102	4%	0.001
Within continents	713	2.178	2.178	84%	0.001	190	2.723	2.723	96%	0.001
Total	717	2.42	2.594			194	2.766	2.826		
VGIII										
Among continents	1	33.565	0.321	12%	0.001	1	4.613	0.082	3%	0.066
Within continents	212	2.318	2.318	88%	0.001	71	2.657	2.657	97%	0.066
Total	213	2.46	2.639			72	2.68	2.739		
VGIV										
Among continents	1	4.217	0.173	8%	0.022	1	2.772	0.057	3%	0.196
Within continents	28	2.036	2.036	92%	0.022	19	2.210	2.210	97%	
Total	29	2.11	2.209			20	2.2	2.267		

df, degrees of freedom; MS, mean square; Est.Var, estimated variance; %, percentage of variance; *p*-value based on 1000 permutations.

**Table 5:** Pairwise population comparison at the continental level.

	Non-Clone-Corrected					Clone-Corrected				
Total CGSC										
	Africa	Asia	Europe	Oceania	N. America	Africa	Asia	Europe	Oceania	N. America
Asia	0.119 ***					0.093 ***				
Europe	0.322 ***	0.220 ***				0.184 ***	0.043 ***			
Oceania	0.410 ***	0.362 ***	0.183 ***			0.053 ***	0.035 ***	0.056 ***		
N. America	0.307 ***	0.255 ***	0.110 ***	0.061 ***		0.120 ***	0.015 ***	0.070 ***	0.075 ***	
S. America	0.223 ***	0.169 ***	0.053 ***	0.128 ***	0.065 ***	0.036 ***	0.026 ***	0.101 ***	0.034 ***	0.034 ***
VGI										
Asia	0.173 ***					0.051 ***				
Europe	0.095 ***	0.210 ***				0.083 ***	0.062 ***			
Oceania	0.453 ***	0.307 ***	0.491 ***			0.403 ***	0.220 ***	0.412 ***		
N. America	0.189 ***	0.104 ***	0.242 ***	0.130 ***		0.153 ***	0.037 ***	0.165 ***	0.123 ***	
S. America	0.145 ***	0.093 ***	0.161 ***	0.237 ***	0.045 ***	0.089 ***	0.010 ***	0.102 ***	0.262 ***	0.045 ***
VGII										
Asia	N/A					N/A				
Europe	N/A	0.191 ***				N/A	0.009 ***			
Oceania	N/A	0.320 ***	0.388 ***			N/A	0.015 ***	0.061 ***		
N. America	N/A	0.229 ***	0.078 ***	0.168 ***		N/A	0.027 ***	0.020 ***	0.066 ***	
S. America	N/A	0.135 ***	0.051 ***	0.158 ***	0.025 ***	N/A	0.00 ***	0.049 ***	0.047 ***	0.051 ***
VGIII										
Asia	N/A					N/A				
Europe	N/A	N/A				N/A	N/A			
Oceania	N/A	N/A	N/A			N/A	N/A	N/A		
N. America	N/A	N/A	N/A	N/A		N/A	N/A	N/A	N/A	
S. America	N/A	N/A	N/A	N/A	0.122 ***	N/A	N/A	N/A	N/A	0.030
VGIV										
Asia	0.078 *					0.025				
Europe	N/A	N/A				N/A	N/A			
Oceania	N/A	N/A	N/A			N/A	N/A	N/A		
N. America	N/A	N/A	N/A	N/A		N/A	N/A	N/A	N/A	
S. America	N/A	N/A	N/A	N/A	N/A	N/A	N/A	N/A	N/A	N/A

* *p* < 0.05; *** *p* < 0.001; N/A, not analyzed due to small sample size.

**Table 6:** Analysis of molecular variance by ecological niche.

Non-Clone-Corrected					Clone-Corrected					
	df	MS	Est.Var	%	*p*-Value	df	MS	Est.Var	%	*p*-Value
Australia										
Among ecological niches	2	23.8	0.32	22%	0.001	N/A	N/A	N/A	N/A	N/A
Within ecological niches	214	1.1	1.14	78%	0.001	N/A	N/A	N/A	N/A	N/A
Total	216	1.36	1.46			N/A	N/A	N/A		
Brazil										
Among ecological niches	2	32.1	0.24	11%	0.001	2	2.99	0.02	1%	0.318
Within ecological niches	400	1.99	1.99	89%	0.001	97	2.66	2.66	99%	0.318
Total	402	2.12	2.24			99	2.67	2.67		
Colombia										
Among ecological niches	2	28.5	0.24	10%	0.001	1	2.73	0.0	0%	0.502
Within ecological niches	332	2.2	2.16	90%	0.001	29	3.03	3.03	100%	0.502
Total	334	3	2.31			30	2.40	3.03		
Taiwan										
Among ecological niches	2	19.2	0.24	13%	0.001	N/A	N/A	N/A	N/A	N/A
Within ecological niches	255	1.58	1.58	87%	0.001	N/A	N/A	N/A	N/A	N/A
Total	257		1.82			N/A	N/A	N/A		
USA										
Among ecological niches	2	29.6	0.26	10%	0.001	1	2.44	0.0	0%	0.54
Within ecological niches	320	2.29	2.29	90%	0.001	45	2.78	2.78	100%	0.54
Total	322	2.46	2.56			46	2.78	2.78		

df, degrees of freedom; MS, mean square; Est.Var, estimated variance; %, percentage of variance; *p*-value based on 1000 permutations. N/A, not analyzed due to small sample size.

**Table 7:** Allelic variation among the seven loci used for the multi-locus sequence typing of the *C. gattii* species complex. The number of shared alleles at each locus between VG lineages.

Gene	Gene Name	Chromosome	Length (bp)	Total Allele Number in CGSC	Number of Shared Alleles between VG Lineages
*CAP59*	Capsular-associated protein	1	556–558	95	1
*GPD1*	Glyceraldehyde-3-phosphate dehydrogenase	7	544–549	61	6
*IGS1*	Ribosomal RNA intergenic spacer	2	624–706	110	4
*LAC1*	Laccase	8	472–475	63	2
*PLB1*	Phospholipase	12	532–535	46	1
*SOD1*	Cu, Zn superoxide dismutase	5	700–713	136	5
*URA5*	Orotidine monophosphate pyrophosphorylase	8	637–639	59	1

**Table 8:** Allelic profiles of sequence types with shared alleles among VG lineages.

ST	No. of Inconsistent ATs	No. of VG Lineages Represented	Originally Assigned VG Lineage	*CAP59*	*GPD1*	*IGS1*	*LAC1*	*PLB1*	*SOD1*	*URA5*
ST11	1	2	VGIV	17	10	2	1	3	93	11
ST53	1	2	VGI	16	5	40	13	5	32	12
ST167	1	2	VGII	2	3	31	4	1	8	7
ST181	1	2	VGII	8	3	22	4	2	87	3
ST223	1	2	VGIII	42	28	61	41	31	51	17
ST335	1	2	VGIII	29	12	11	9	13	28	22
ST350	1	2	VGII	5	11	25	4	16	16	6
ST351	4	2	VGI	44	16	59	4	13	14	14
ST355	1	2	VGII	3	5	57	4	18	58	2
ST356	1	2	VGI	16	6	3	5	5	45	12
ST357	1	2	VGII	1	6	25	4	2	45	7
ST386	1	2	VGIV	63	11	2	1	3	62	11
ST400	1	2	VGII	2	52	56	21	1	90	7
ST402	6	3	VGIV	25	11	46	13	13	73	11
ST408	1	2	VGI	71	42	90	6	5	113	38
ST409	1	2	VGII	2	52	56	21	1	8	7
ST419	1	2	VGIV	23	4	2	17	7	35	34
ST431	2	3	VGI	44	4	59	13	13	68	15
ST451	1	2	VGII	8	6	14	4	2	6	3
ST508	1	2	VGIV	23	4	103	17	44	35	34
ST524	2	3	VGI	44	4	102	13	13	68	15
ST528	1	2	VGI	71	56	90	6	5	113	38
ST550	1	2	VGI	16	5	12	5	5	32	3

No., number; Pink represents the VGI lineage, light blue represents the VGII lineage, dark blue represents the VGIII lineage, yellow represents the VGIV lineage, and orange represents STs inconsistently grouped in Figure 1.

**Table 9:** Summary of genotypic diversity and phylogenetic incompatibility based on the sequence type and VG lineage information assigned by the MLST database. ** *p* < 0.01; *** *p* < 0.001.

Population	Number	Phylogenetic Compatibility (% of 21 Pairs)	Index of Association
Global CGSC	566	0	0.82 ***
Global VGI	160	0	1.27 ***
Global VGII	218	0	0.28 ***
Global VGIII	75	9.5%	1.46 ***
Global VGIV	111	23.8%	0.14 **
Africa CGSC	31	52.4%	2.10 ***
Asia CGSC	50	71.4%	1.99 ***
Europe CGSC	46	76.1%	2.40 ***
Oceania CGSC	34	90.4%	3.10 ***
North America CGSC	94	14.2%	1.96 ***
South America CGSC	174	0	0.55 ***
Asia VGI	29	76.1%	1.63 ***
Europe VGI	28	85.7%	1.51 ***
Oceania VGI	21	90.4%	1.05 ***
North America VGI	23	90.4%	1.43 ***
North America VGII	59	19%	1.37 ***
South America VGII	148	0	1.60 ***

## Data Availability

All data analyzed in this study are cited and are summarized in the manuscript.

## Supplementary Materials

The following supporting information can be downloaded at: https://www.mdpi.com/article/10.3390/jof9020276/s1, Figure S1: Phylogenetic tree at *CAP59*; Figure S2: Phylogenetic tree at *LAC1*; Figure S3: Phylogenetic tree at *IGS1*; Figure S4: Phylogenetic tree at *PLB1*; Figure S5: Phylogenetic tree at *SOD1*; Figure S6: Phylogenetic tree at *URA5*; Table S1: Ecological distribution of CGSC sequence types; Table S2: Ecological distribution of CGSC molecular types; Table S3: Continent distribution of CGSC sequence types; Table S4: Country distribution of CGSC sequence types; Table S5: Pairwise geographic population genetic differentiation of the total CGSC sample; Table S6: Pairwise geographic population genetic differentiation of the total VGI sample; Table S7: Pairwise geographic population genetic differentiation of the total VGII sample; Table S8: Pairwise geographic population genetic differentiation of the total VGIII sample; Table S9: Pairwise geographic population genetic differentiation of the total VGIV sample; Table S10: Pairwise comparison among ecological niches; Table S11: Occurrence of allele types at each of the seven loci among the 566 STs; Table S12: Linkage disequilibrium analyses results.
