# Supplementary material for: Global Analyses of Multi-Locus Sequence Typing Data Reveal Geographic Differentiation, Hybridization, and Recombination in the Cryptococcus gattii Species Complex
Source: J Fungi (Basel). 2023 Feb 20;9(2):276. doi: 10.3390/jof9020276 (PMC9967412; doi:10.3390/jof9020276)
Supplement: Supplementary file 1 [file jof-09-00276-s001.zip › Supplementary Figures.pdf]

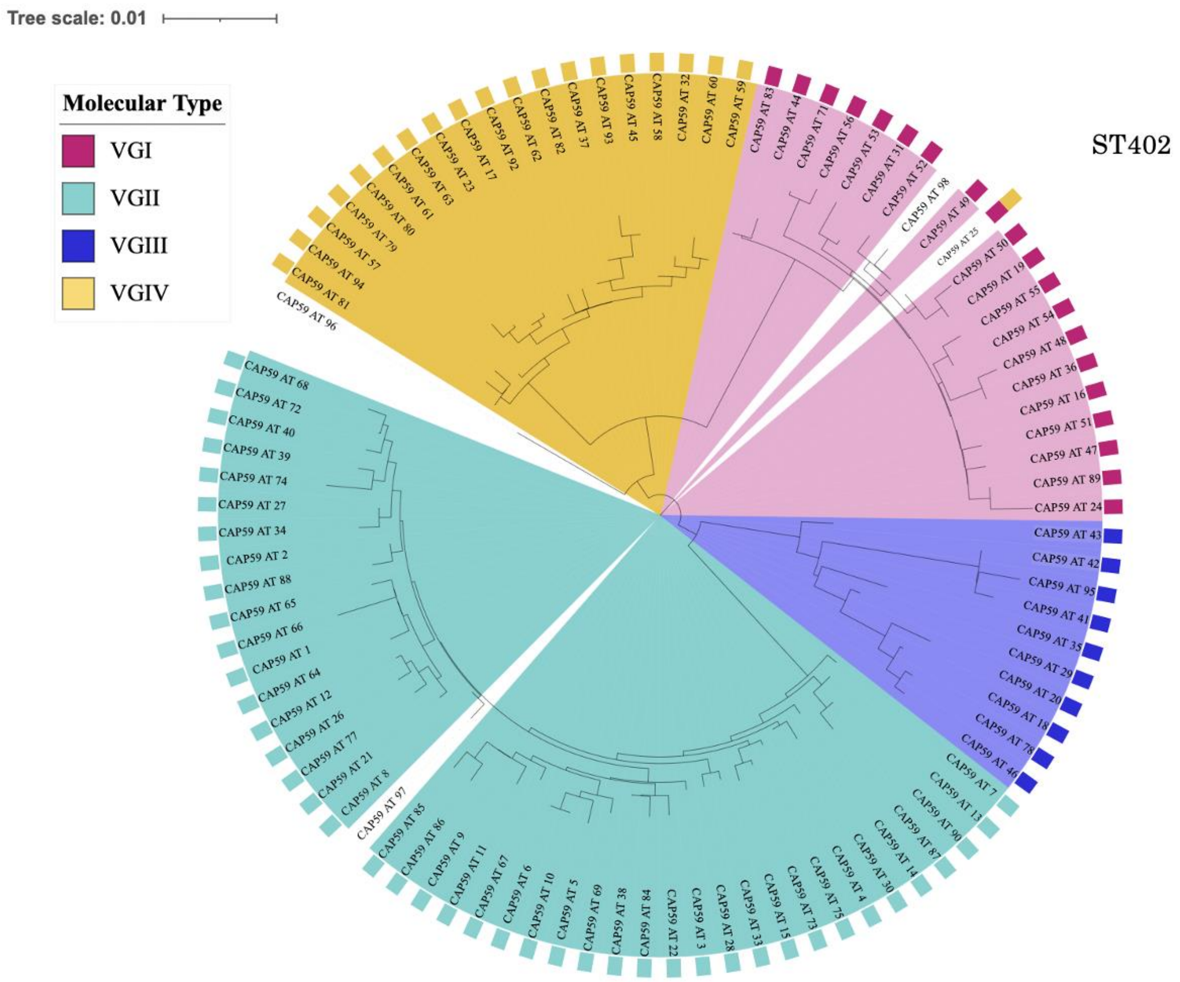

SF1: Phytogenic tree illustrating the relationship among allele types (AT) found at the *CAP59* locus of the C. *gattii* species complex. Four colours are used to display which lineage each AT is recorded in. Legends on the left indicate which colour corresponds to which lineage.

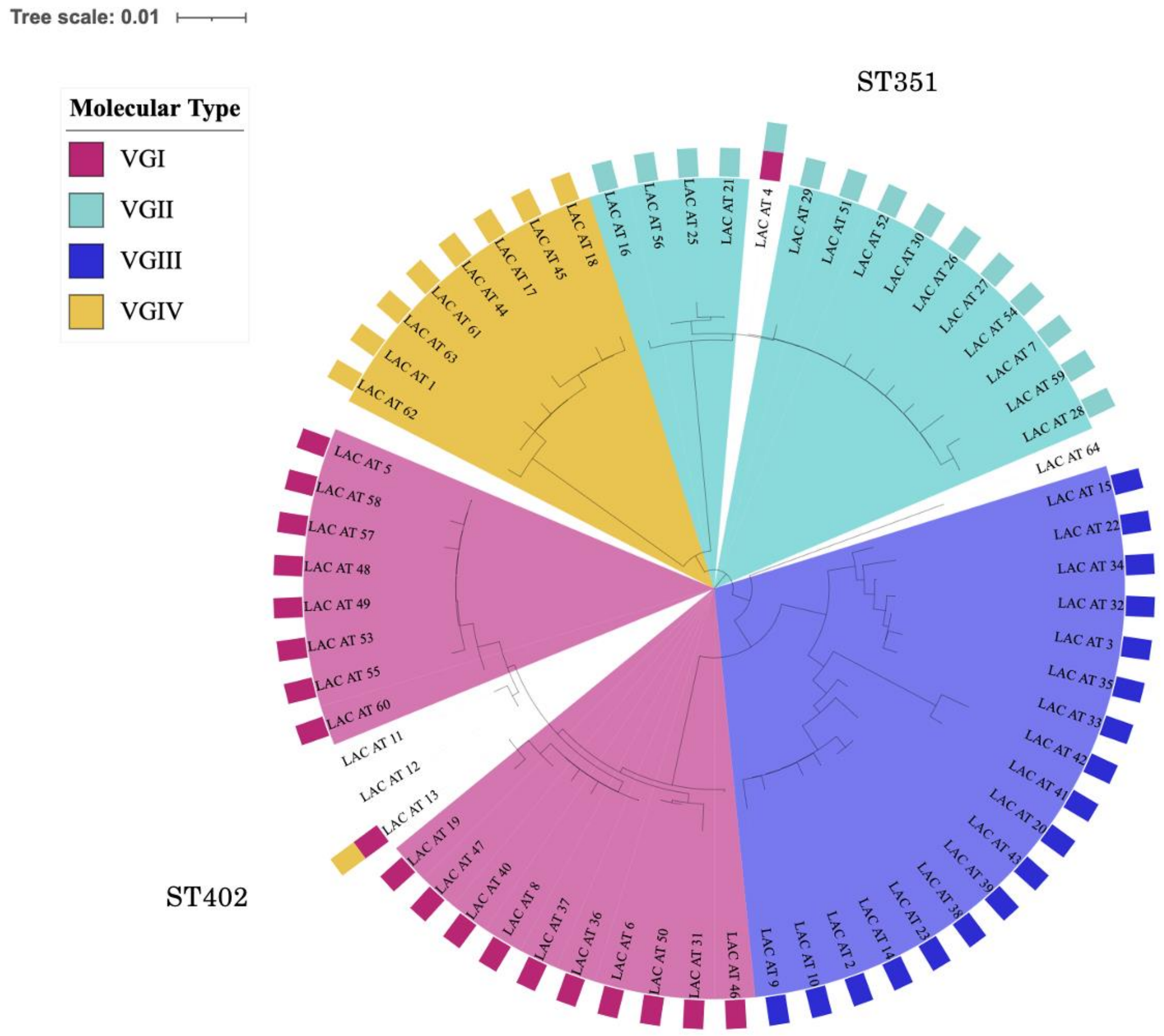

SF2: Phytogenic tree illustrating the relationship among allele types (AT) found at the *LAC1* locus of the C. *gattii* species complex. Four colours are used to display which lineage each AT is recorded in. Legends on the left indicate which colour corresponds to which lineage.

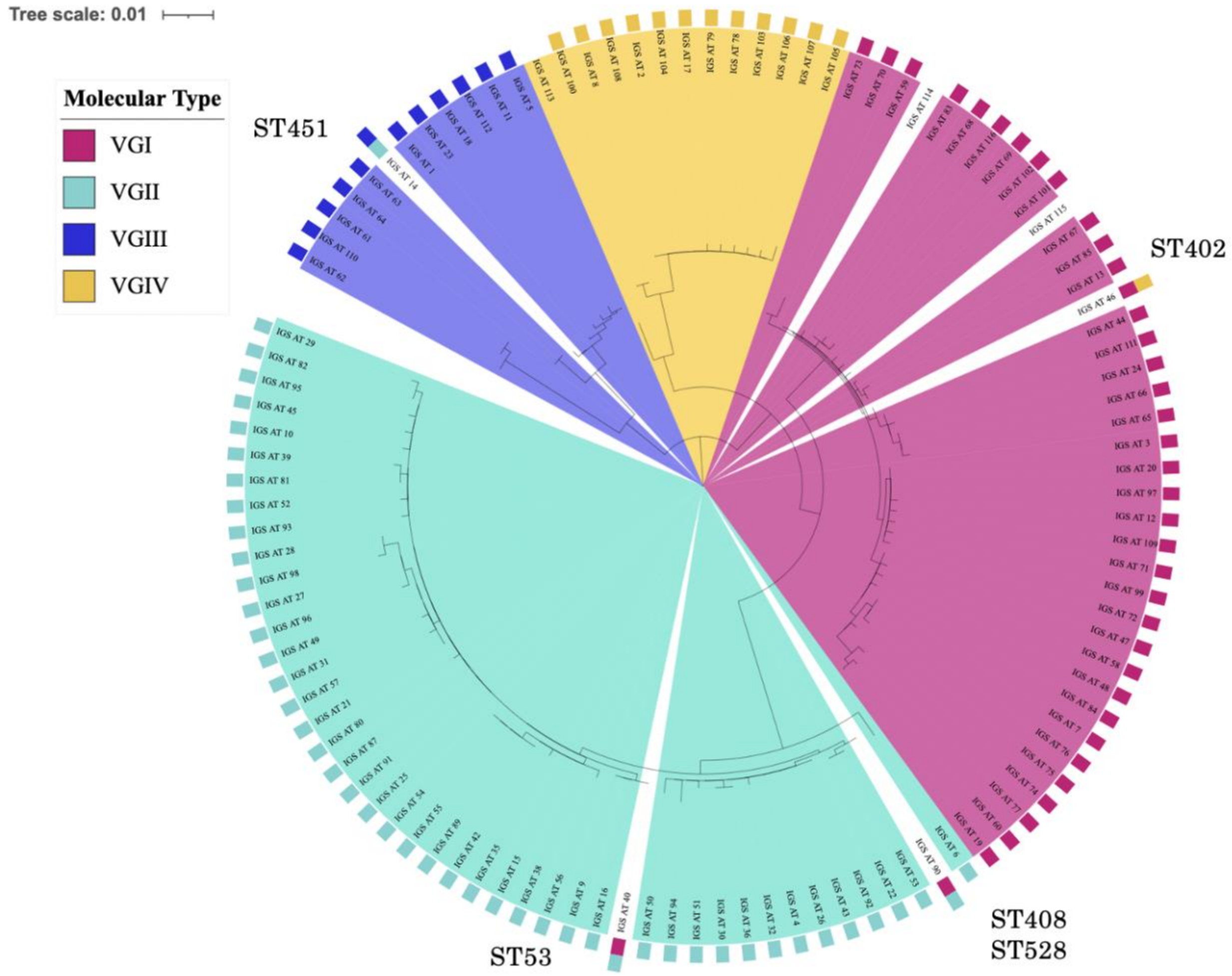

SF3: Phytogenic tree illustrating the relationship among allele types (AT) found at the *IGS1* locus of the *C. gattii* species complex. Four colours are used to display which lineage each AT is recorded in. Legends on the left indicate which colour corresponds to which lineage.

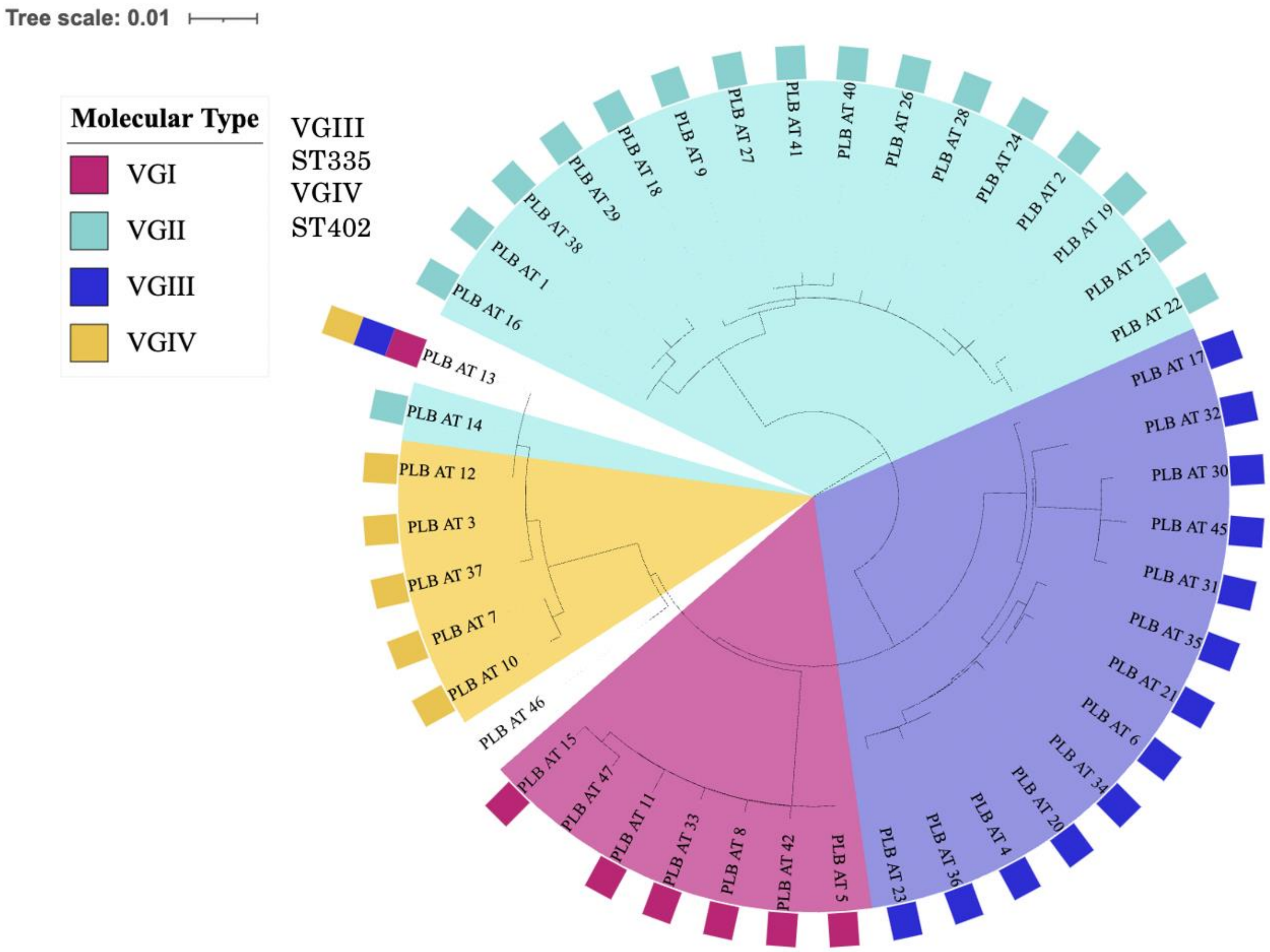

SF4: Phytogenic tree illustrating the relationship among allele types (AT) found at the *PLB1* locus of the C. *gattii* species complex. Four colours are used to display which lineage each AT is recorded in. Legends on the left indicate which colour corresponds to which lineage.

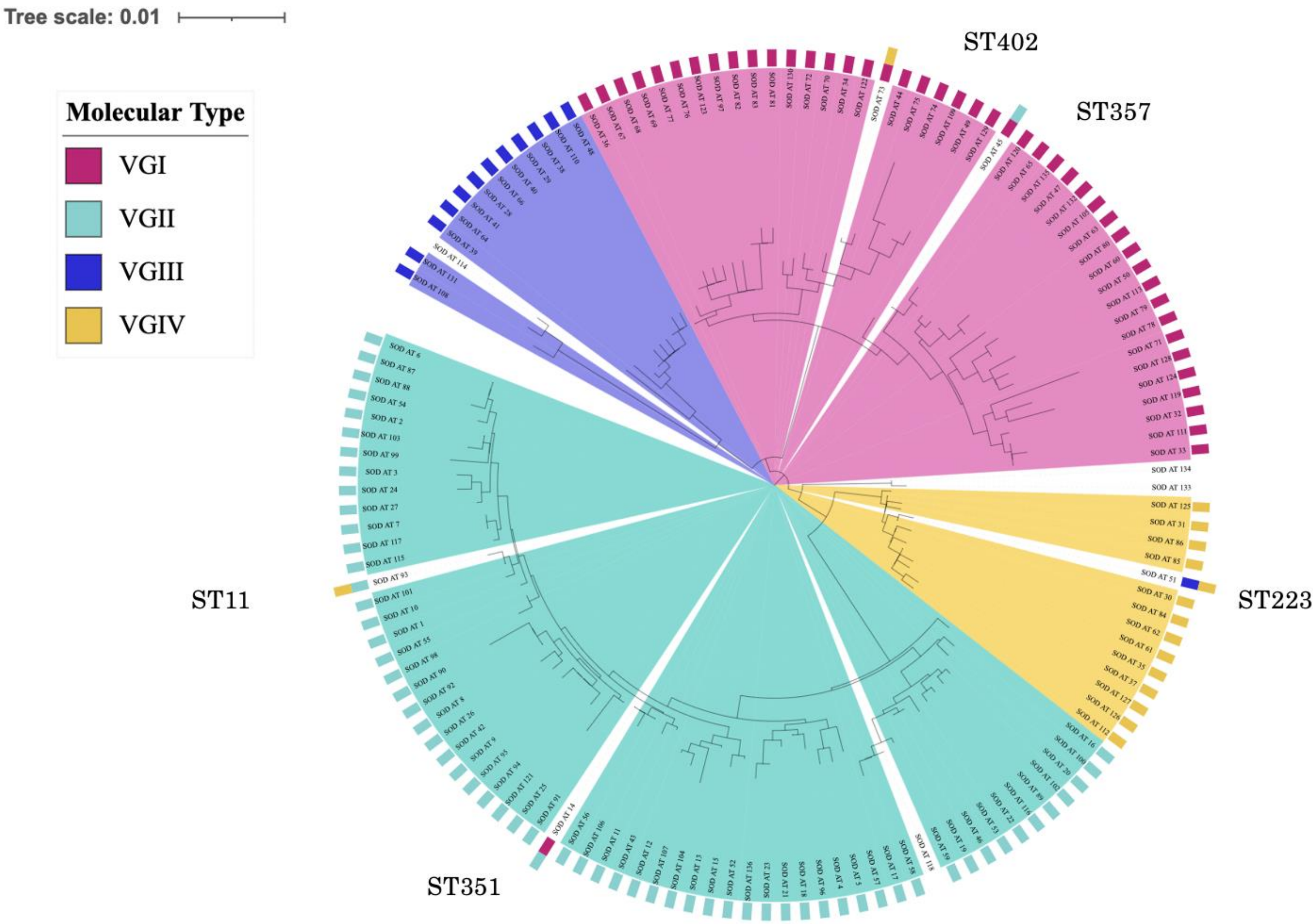

SF5: Phytogenic tree illustrating the relationship among allele types (AT) found at the *SOD1* locus of the C. *gattii* species complex. Four colours are used to display which lineage each AT is recorded in. Legends on the left indicate which colour corresponds to which lineage.

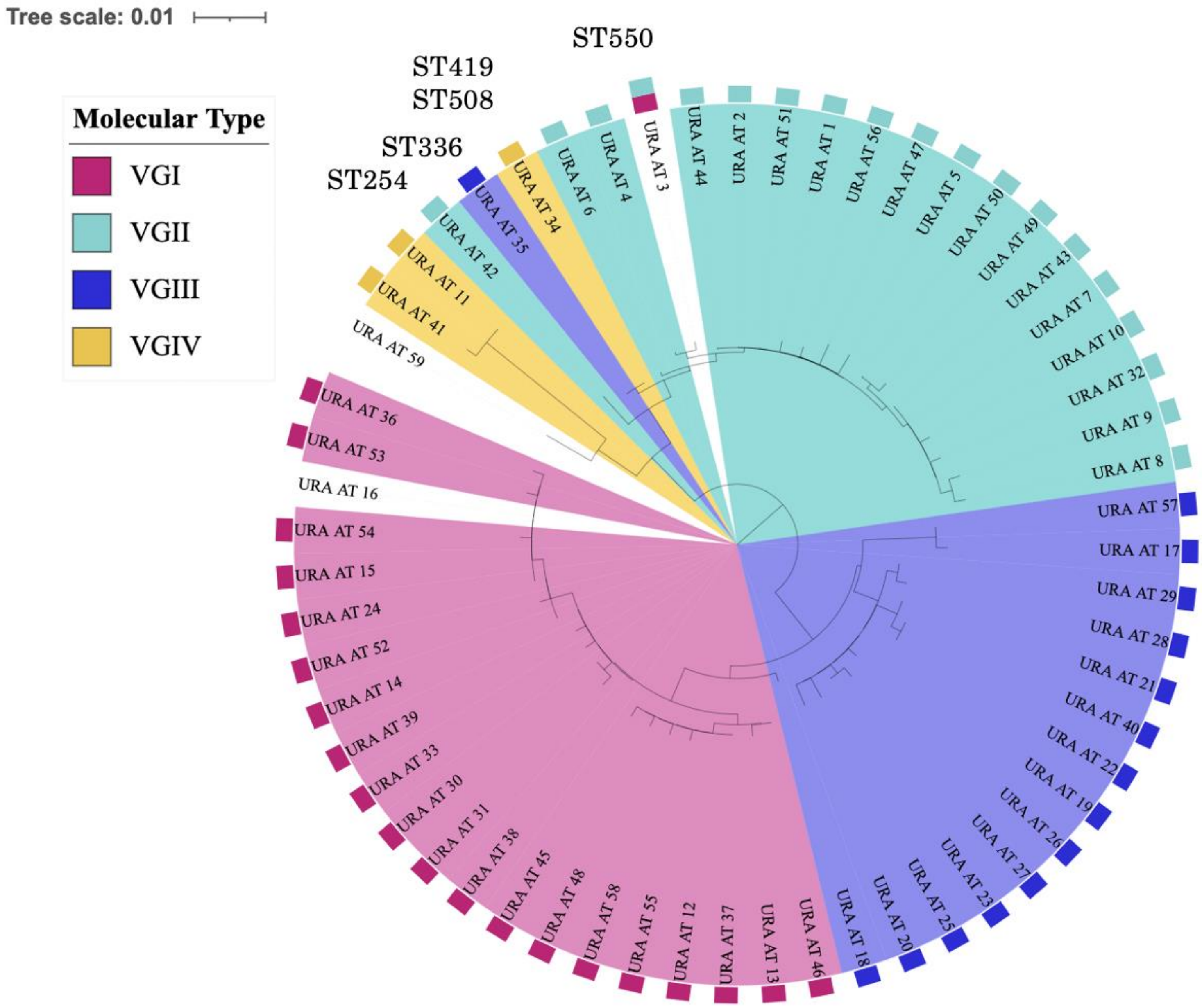

SF6: Phytogenic tree illustrating the relationship among allele types (AT) found at the *URA5* locus of the C. *gattii* species complex. Four colours are used to display which lineage each AT is recorded in. Legends on the left indicate which colour corresponds to which lineage.
